# Risk stratification in diabetic ankle fractures: a systematic review of reviews and proposal of the MADRAS scoring system

**DOI:** 10.1186/s40842-026-00292-6

**Published:** 2026-07-01

**Authors:** Ahmed Bakr, Maher Nassor, Usman Muhammad, Marwan Tahoun, Anand Pillai

**Affiliations:** 1 University of Liverpool School of Medicine, Liverpool, UK; 2 University of Sunderland School of Medicine, Sunderland, UK; 3 Warrington and Halton Hospitals NHS Foundation Trust, Warrington, UK; 4 Manchester University NHS Foundation Trust, Manchester, UK; 5 University of Manchester, Manchester, UK

**Keywords:** Diabetic ankle fracture, Risk stratification, MADRAS, AFDA, Systematic review, Peripheral neuropathy, Peripheral arterial disease, ORIF, Tibiotalocalcaneal arthrodesis, Charcot neuroarthropathy

## Abstract

**Background:**

Diabetic ankle fractures present significant clinical challenges, with higher rates of complications including infection, non-union, Charcot neuroarthropathy, and amputation compared to non-diabetic patients. Despite this burden, no universally accepted guidelines exist for risk stratification and management. The Adelaide Fracture in the Diabetic Ankle (AFDA) score was proposed in 2014 but has limitations including uniform weighting of risk factors and exclusion of local injury characteristics. This systematic review of reviews aimed to synthesise current evidence and develop an updated, evidence-based risk stratification tool.

**Methods:**

A comprehensive search of Embase, PubMed, and Web of Science was conducted. Eligible studies included systematic and narrative reviews examining outcomes, risk factors, or interventions for diabetic ankle fractures. Risk of bias was assessed using the ROBIS tool. Data were synthesised narratively, and an evidence-informed, consensus-based scoring system was developed by translating observed effect sizes from the included reviews into weighted risk scores.

**Results:**

Thirteen reviews were included. Complicated diabetes, particularly peripheral neuropathy, peripheral arterial disease, and prior Charcot neuroarthropathy, emerged as the strongest predictors of adverse outcomes. Non-operative management of unstable fractures was associated with significantly higher complication rates. Based on these findings, we propose the Manchester Ankle Fracture Diabetic Risk Assessment Score (MADRAS), incorporating weighted systemic host factors, local injury characteristics, and modifiable patient factors across a 17-point scale stratifying patients into four management tiers: low risk (0–2), moderate risk (3–5), high risk (6), and very high risk (≥ 7).

**Conclusion:**

MADRAS addresses limitations of existing frameworks by incorporating evidence-based weightings and local injury factors. Prospective external validation is required before widespread clinical adoption; until then, MADRAS should be regarded as a decision-support tool. This tool may assist clinicians in systematic risk stratification and surgical decision-making for diabetic ankle fractures.

## Background

Diabetes mellitus is a major and rapidly growing global health challenge, with prevalence increasing steadily over recent decades; the number of people living with diabetes has increased from 200 million in 1990 [1] to 590 million in 2024, with projections estimating this figure to rise to 853 million by 2050 [2]. Fracture risk is significantly higher due to compromised bone microarchitecture, accumulation of advanced glycation end products (AGEs) in the bone matrix, and reduced bone turnover, all of which negatively affect bone strength and quality [3–5]. A notable consequence of diabetes is peripheral neuropathy, which occurs due to chronic hyperglycaemia and insulin resistance, which when combined induce metabolic and vascular complications in peripheral nerves. It is seen in up to 50% of diabetes patients [6]. Patients with this have also been shown to be at a higher risk of falls, due to loss of sensation in lower limbs leading to poor balance [7]. The ankle and foot are of particular concern in these patients due to higher risk of ulceration, infection, Charcot foot, and increased risk of fractures, primarily as a result of peripheral neuropathy, peripheral artery disease, and structural foot deformities; all of which can result in tissue breakdown and impaired healing [8]. As a result, diabetic patients experience poorer outcomes following foot and ankle fractures compared to non-diabetic people, with higher rates of complications including infection, non-union, malunion, and re-operation [9]. Higher healthcare utilisation has also been reported after management of such fractures in these patients [10]. Given the rising global burden of diabetes and the significant consequences of lower limb fractures in these patients, understanding this link is becoming increasingly important within healthcare.

Despite the recognised burden of diabetic foot and ankle fractures, current clinical practice lacks consistency and management approaches vary widely. The management of these fractures is complicated by factors such as neuropathy, vascular disease, poor bone quality, and impaired healing which necessitate individualised treatment plans. For unstable fractures, rigid internal fixation, soft-tissue management and prolonged immobilisation are recommended according to current literature [11], whilst for nondisplaced fractures, non-operative management with extended immobilisation may be appropriate [12]. There are also no universal guidelines or standardised protocols for the diagnosis and management of diabetic foot fractures, and treatment decisions are based on individual patient comorbidities, fracture characteristics and clinician discretion. Such variability can lead to misdiagnosis, inefficient management, and increased healthcare utilisation in this population [13].

Given the substantial burden of diabetic foot and ankle fractures and the variability in diagnostic and treatment approaches [13], there is a clear need for a comprehensive synthesis of the existing evidence. A systematic review of reviews offers the methodological advantage of integrating findings from multiple systematic reviews, providing clinical decision makers with a comprehensive summary of the evidence base, allowing for a broader evaluation of outcomes, risk factors and the relative effectiveness of available interventions [14]. This review aims to highlight areas of consensus, highlight persistent uncertainties, and provide clinicians with an accessible, comprehensive overview to support informed decision making. Such synthesis is invaluable to guide future research and contribute towards more standardised and effective management pathways for diabetic foot and ankle fractures.

Therefore, the aims of this systematic review of reviews are twofold: first, to synthesise the existing evidence on diabetic foot and ankle fractures by appraising the clinical outcomes associated with these injuries, the risk factors that contribute to fracture-related complications, and the effectiveness of available management interventions; and second, to propose an updated, evidence-based risk stratification tool—the Manchester Ankle Fracture Diabetic Risk Assessment Score (MADRAS)—that addresses the limitations of the existing AFDA framework by incorporating weighted risk factors based on their observed effect sizes and including local injury characteristics alongside systemic host factors.

## Methods

### Information sources and search strategy

A comprehensive search was conducted across Embase via Ovid, PubMed, and Web of Science on 09/11/2025. A search strategy was developed in advance for locating the studies that fit the inclusion criteria, including controlled headings and synonyms to capture all the possible studies for inclusion in all three databases.

### Eligibility criteria

Eligible studies included in this review were comprised of reviews such as systematic reviews or narrative reviews that focused on outcomes, risk factors, or optimal interventions of diabetic foot ankle fractures. Studies that compared different interventions, or one in isolation, were included. Outcomes-specific reviews to diabetic foot fracture interventions, regardless of the type of interventions, were eligible for inclusion. Despite the interventions, outcomes, or evaluation of risk factors, primary studies were excluded.

### Study selection

All studies retrieved from the database searches were imported to Rayyan, and duplicate studies were subsequently removed. One reviewer (MN) screened the titles and abstracts to identify potential studies for inclusion, followed by full-text screening to validate eligibility against the predefined criteria. All screened articles were further reviewed by other reviewers (AB and UM) to check if all the included studies had met the inclusion criteria before the data extraction commenced.

### Data extraction

Data extraction was conducted independently using the prespecified template by two reviewers (AB and UM). Extracted information included all relevant outcomes of Diabetic foot ankle fracture interventions, as well as risk factors leading to diabetic foot ankle fracture. Any misalignment was resolved through discussion with the third reviewer (MT).

### Risk of bias assessment

The quality of the included studies was assessed using the Risk of Bias in Systematic reviews (ROBIS). This is because the ROBIS evaluates key domains such as study eligibility criteria and data collection, and study appraisal. The decision to employ ROBIS was made in accordance with the inclusion criteria, allowing only reviews to be evaluated for this systematic review. Risk of bias assessment was conducted independently using the ROBIS tool by one reviewer (UM) and cross-checked by a second reviewer (AB) to ensure consistency of assessment. Any discrepancies were resolved through discussion with a third reviewer (MT).

Once the risk of bias assessment was complete, the results were visualised using the Risk of Bias Visualisation (ROBVIS) tool. This enabled a clear presentation of study ratings.

### Data synthesis and statistical analysis

Given the nature of this systematic review, data were synthesised narratively. Any meta analytic data was summarised descriptively and patterns in outcomes, interventions, and risk factors of diabetic foot ankle was synthesised and highlighted accordingly. Based on the synthesised findings, a risk stratification tool was developed by translating the observed effect sizes from the included reviews into weighted scores through structured consensus among the author group; risk factors with OR ≥ 10 received 3 points, those with OR 3–10 or complication rates exceeding 50% received 2 points, and those with OR < 3 received 1 point. Where no pooled OR was available for a given risk factor, weighting was assigned on the basis of reported complication rates (applying the same thresholds) or, where neither OR nor complication rate data were reported in the included reviews, on the basis of expert clinical consensus among the author group; such instances are explicitly identified in Tables 2, 3 and 4. This approach is evidence-informed and consensus-based rather than statistically derived through regression modelling [15].

## Results

**Fig. 1:**
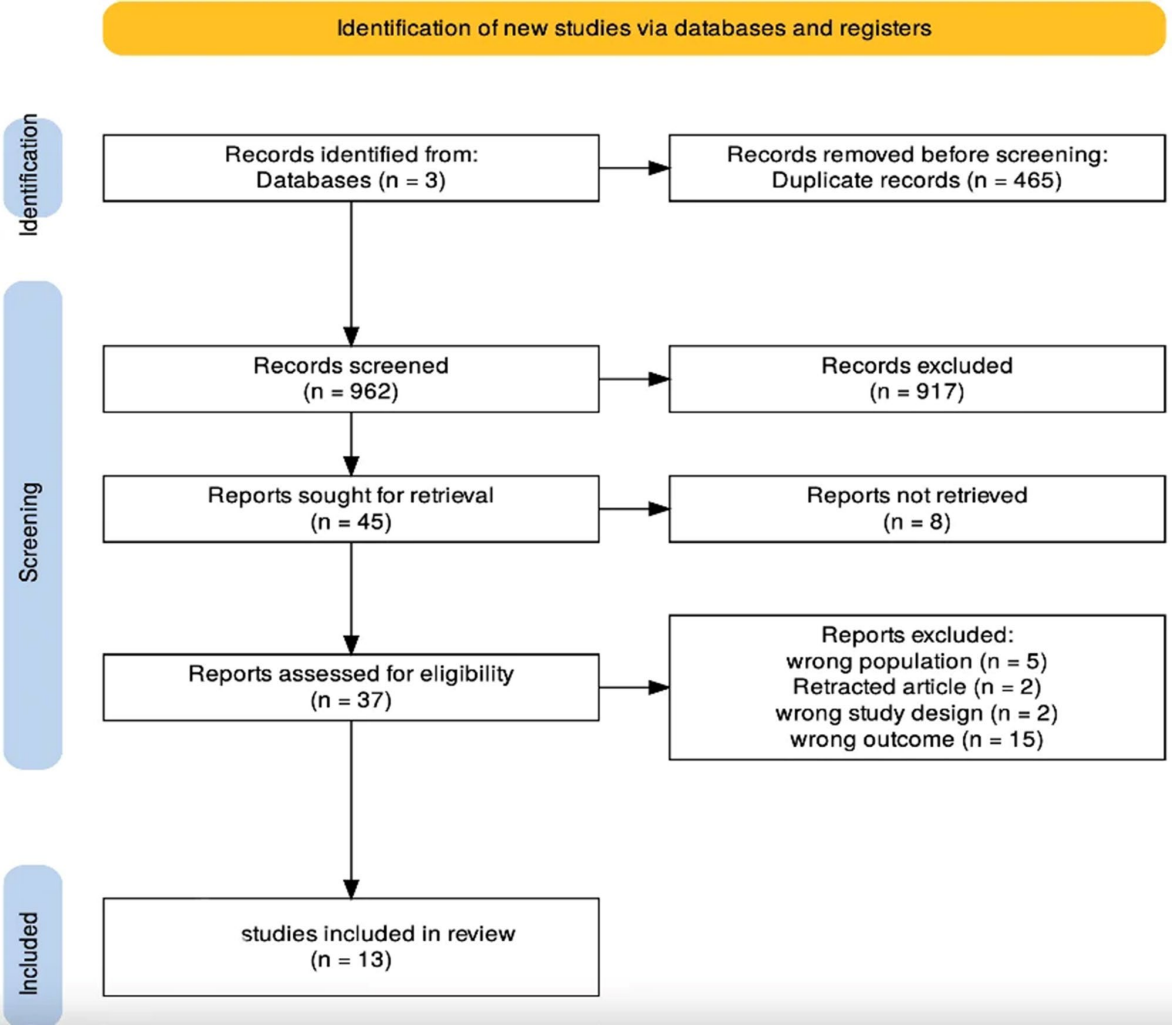
PRISMA flow diagram showing the results of study identification, screening, and final included studies. A total of 13 reviews met inclusion criteria after screening of titles, abstracts, and full texts from Embase, PubMed, and Web of Science databases

The study selection process is presented in Fig. 1.

Characteristics of the 13 included reviews are summarised in Table 1.

**Table 1:** Characteristics of included reviews. Summary of study type, patient details, risk factors, interventions, and outcomes for all 13 included reviews examining diabetic ankle fractures

Study	Study Type	Patient Details	Risk Factors	Interventions	Outcomes
Diabetic ankle fractures complications: a meta-analysis López et al. (2020)	SR and MA (12 observational studies)	220,167 patient’s total; 16,910 with diabetes vs. 203,257 without; mean age 51.4; 36% insulin-dependent; 64% non-insulin-dependent	Diabetes itself increases complication risk after ankle fracture. Advanced / complicated DM (neuropathy, vasculopathy, poor control, nephropathy/retinopathy) is the main driver: OR 8.4 vs. non-complicated DM. Severe complications (deep infection, Charcot/non-union, amputation) cluster in patients with advanced diabetic disease.	Emphasises early identification of high-risk ankles and optimisation of diabetes. Proposes CAMPA (Charcot, Anamnesis of advanced disease, Monofilament, Pulses, A1c) as a screening tool to flag high-risk diabetic ankle fractures.	Any complication: OR 1.9 (DM vs. non-DM). Infection overall: OR 3.4; deep infection: OR 9.9. Charcot/malunion/non-union: OR 5.1. Amputation ≈ 5% in diabetics; OR 2.9 (wide CI). Mortality: OR 3.5. In surgically treated ankles, complications ≈ 4× higher in diabetics: OR 3.7.
A systematic review of ankle fracture treatment modalities in diabetic patients Manchanda et al. (2020)	Systematic review	420 operative ankle fractures; mean age 57.9; 45.4% male; follow-up 21.7 months.	Neuropathy repeatedly reported in pts requiring combined constructs; high BMI and nephropathy contributed to hardware failure in some cases.	Standard ORIF (plates/screws) Minimally invasive (percutaneous screws, fibular/TTC nails) Combined constructs (ORIF + trans-articular screws/ex-fix): used in very high-risk feet	Pooled across 420 fractures: infection 14.3%, wound problems 10.2%, re-operation 16.7%, non-union 3.8%, Charcot 3.3%, limb salvage 97.9%. Minimally invasive vs. standard: similar limb salvage (~ 98%), fewer infections (~ 7.5% vs. 15%) but more hardware breakage (~ 5% vs. 0.7%). Combined constructs vs. standard: infection 35.7%, non-union 14.3%, limb salvage 85.7% vs. 98.2%
Ankle fractures in diabetic patients Gougoulias et al. (2020)	Narrative review	Summarised data from multiple cohorts ~ 13% of all ankle fixation pts are diabetic; ~2% have complicated DM	Complicated DM (neuropathy, PAD, nephro/retinopathy, poor HbA1c) strongly increases infection, non-union and amputation risk. Uncomplicated DM behaves similarly to non-diabetic ankles; excess risk is driven by complicated/neuropathic cases. HbA1c > 6.5–8% and neuropathy repeatedly associated with poorer ankle fracture outcomes.	Non-operative treatment of unstable fractures in complicated DM is discouraged. Recommends rigid fixation (locking plates, multiple screws, super-constructs) in high-risk patients. Primary arthrodesis (TTC nail / frame / plate fusion) is an option for severe, unstable or failed cases.	Overall complication rates up to 47% in diabetics vs. ~ 15% in non-diabetics. SSI ≈ 13.2% vs. 2.8%; deep infection ≈ 6.9% vs. 1.3% (DM vs. non-DM). Arthrodesis series in diabetics show high fusion and limb-salvage rates (~ 80–96%) in complex cases.
Primary arthrodesis for diabetic ankle fractures Grote et al. (2020)	Retrospective case series	13 diabetic pts; mean age 67.1; 46% male; mean BMI 35.8; mean follow-up 297 days. 77% insulin-dependent; mean HbA1c 7.5 Severe comorbidities: Neuropathy 81.8% PVD 61.5% Nephropathy 69.2% Retinopathy 46.2% Prior Charcot 15.4% Mean 2.8 complications; mean AFDA score 6.4 Injury patterns: - 69.2% fracture dislocations - 38.5% open fractures	Very high-risk cohort: mean AFDA 6.4, most with neuropathy, PAD, nephropathy, retinopathy and multiple diabetic complications. Injuries were severe: majority fracture–dislocations and a high proportion of open fractures	All patients treated with primary TTC hindfoot nail, often functioning as a rigid internal fixator rather than a true fusion construct. TTC nailing used as a limb-salvage strategy in patients with high AFDA scores and severe injury patterns.	Overall complications 76.9%; infection 38.5%; wound problems 53.8%; hardware failure 30.8%; re-operation 38.5%. Arthrodesis non-union 77.8%, but fracture union 89%, suggesting TTC nail succeeded mainly as an internal fixator. Amputation 23.1%; Charcot 7.7%; 1 postoperative death.
Diabetic ankle fractures: a review of the literature Yee et al. (2014)	Narrative review with development of a new coding system (AFDA)	This paper does not present a cohort. Synthesis of prior studies to develop risk stratification	Identifies neuropathy, PAD, nephropathy, retinopathy, poor glycaemic control, long-standing DM, prior ulcer/Charcot and deformity as key risk factors. Distinguishes uncomplicated vs. complicated diabetes and notes much higher complications in the latter.	Introduction of Adelaide Fracture in the Diabetic Ankle (AFDA) score Low AFDA scores (0–4): manage similarly to non-diabetics with standard ORIF. Intermediate/high AFDA scores (≥ 5): recommend augmented fixation or primary arthrodesis, and prolonged protection; avoid non-operative treatment in neuropathic/complicated DM.	Diabetic ankles show higher rates of infection, ulceration, non-union, malunion, Charcot and amputation than non-diabetics. Deep infection reported in 6–20%; re-operation in 20–35% of diabetic ankles. Amputation risk increased in patients with complicated diabetes.
Early weight bearing after ankle fracture surgery: a systematic review and meta-analysis of functional outcomes and safety Wang C et al. (2025)	Systematic review and meta-analysis (12 studies, 1847 participants)	Adults 18 or over with operatively treated ankle fractures Various fracture types (SER/Weber A-C) Diabetes reported in 6/12 studies, with some trials excluding poorly controlled diabetes (HbA1c > 8.5%)	Diabetic subgroup had worse recovery with delayed protocols, but showed greater benefit with early weight-bearing Improved OMAS (+ 18.3 at 12 weeks) Faster ADL return (9.1 vs. 12.8 weeks) Lower complication rates (11.2% vs. 18.7%)	Compared early weight-bearing (≤ 2 weeks) with delayed weight-bearing (> 2–6 weeks) after ORIF In patients with syndesmotic fixation, older age or diabetes, authors recommend slightly prolonged non-weight-bearing (extra 1–4 weeks) and individualised protocols.	Functional: early weight bearing (≤ 2 weeks) improved pain (SMD + 0.32) and dorsiflexion (SMD + 0.38) Faster return to activities: return to work 12.3 weeks earlier 75% of EWB pts reached ROM > 85% of normal by 12 weeks (vs. 40% DWB) Radiographic: higher union rates with early weightbearing (78% vs. 56% by week 16) Improved reduction maintenance (76.5% vs. 63%) Complications: overall risk similar (RR 0.89). Fewer immobilisation complications
Surgical aspects of the diabetic foot Robinson et al. (2009)	Narrative review	Synthesis of evidence on neuropathy, vasculopathy, ulceration, Charcot and fractures.	Peripheral neuropathy and absent pulses/PAD strongest predictors of complications in diabetic ankle fractures. Long-standing diabetes, nephropathy, retinopathy, prior Charcot and poor glycaemic control further increase risk. Previous Charcot deformity is associated with particularly poor prognosis after ankle fracture.	Stable fractures: may be managed non-operatively but with prolonged immobilisation and careful monitoring. Unstable fractures without critical ischaemia: recommend operative fixation with rigid constructs and double usual immobilisation time in neuropathic patients. In severe PAD, revascularisation should precede fixation; if revascularisation is impossible, non-operative care may be the only option.	Diabetic ankle fractures have a high complication rate, with nonunion, loss of fixation, malunion, wound problems and severe deformity from secondary Charcot neuroarthropathy commonly reported. In one series, 10/12 patients with absent foot pulses developed complications (≈ 83%) Non operative mgmt. associated with higher rates of failure, delayed collapse, Charcot related deformity. Operative mgmt. complication rate 14% less than non-operative. Poor prognosis in pts with hx of Charcot
Current concepts and challenges in managing ankle fractures in the presence of diabetes: a systematic review of the literature Nash et al. (2021)	Systematic review	Mixed populations of diabetic pts with acute ankle fractures; includes unstable and stable fractures, open and closed patterns, neuropathic and non-neuropathic groups. Subgroups include IDDM vs. NIDDM and complicated vs. uncomplicated DM	Complicated diabetes: 47.6% complication rate vs. 14.3% in uncomplicated diabetes. Strong independent risk factor for infection (OR ~ 12) Neuropathy is the single strongest predictor (OR ≈ 15 for SSI in one series). IDDM, PVD, open fractures and poor compliance further increase risk; uncomplicated DM approximates non-DM in some series	Non operative mgmt.: should not be used for unstable fractures. Operative: early fixation recommended; no increase in complications Rigid fixation (“ORIF plus”): multiple syndesmotic screws, fibula-pro-tibia technique. External fixation: use with caution Deep infection management: retaining hardware until fracture union is recommended. Salvage: hindfoot arthrodesis or revision ORIF preferred over closed	Complications up to 30% in diabetics vs. ~ 75 in non-diabetics Neuropathy: OR 15.49 for major complications; strongly linked to infection, non-union, Charcot Non-operative unstable fractures: 75% complications; secondary surgery after failed casting = 100% complications Amputation risk higher in diabetics, especially IDDM Early weight-bearing: acceptable in stable, non-neuropathic fractures; neuropathic patients = ~ 50% complication rate Costs: DM increases hospital LOS + cost; complicated diabetes increases both. ORIF plus (fibula-pro-tibia technique): complication rate 5.7% vs. 20% with standard ORIF.
Do patients with diabetes have an increased risk of impaired fracture healing? A systematic review and meta-analysis Ding et al. (2020)	Systematic review and meta-analysis	695 pts with diabetes 4937 controls 10 lower extremity studies (incl. 3 ankle fracture studies), 2 upper extremity, 2 multiple-site cohorts Diabetes types analysed separately (IDDM vs. NIDDM) Mixed surgical and non-surgical fractures	Types of DM: both IDDM (OR 4.04) and NIDDM (5.83) assoc. with significantly higher risk of impaired healing. No diff. between types Lower extremity vulnerability: neuropathy, PAD poor glycaemic control (high HbAA1c) linked to impaired healing in several studies Comorbidities: peripheral neuropathy, nephropathy, vascular disease contribute to impaired healing	No direct treatment interventions evaluated.	Overall impaired healing risk: OR 2.11 (95% CI 1.33–3.37); diabetics have 2x risk of impaired fracture healing Lower extremity fractures: OR 2.63 (95% CI 1.30–5.30) Short bone fractures (ankle, hand, foot): OR 2.64 (95% CI 1.35–5.20)
Fractures and dislocations of the foot and ankle in people with diabetes: a literature review Johnson et al. (2023)	Narrative literature review	Adults with diabetes sustaining foot or ankle fractures (multiple cohort studies and case series). Diabetes associated with 32% higher relative risk of all fractures and 24% higher relative risk of ankle fractures vs. non-diabetics. Type 2 diabetes associated with 37% higher relative risk of foot fractures vs. non-diabetics. Distinguishes uncomplicated diabetes from complicated diabetes (neuropathy, peripheral arterial disease, chronic renal disease).	Complicated DM (neuropathy, PAD, nephropathy, retinopathy, poor glycaemic control) strongly predicts infection, non-union and amputation. Previous Charcot, ulceration and severe soft-tissue compromise further increased risk. Uncomplicated DM has outcomes closer to non-diabetics when managed appropriately	Uncomplicated DM: treat fractures similarly to non-diabetics with standard ORIF if soft tissues permit. Complicated/neuropathic DM: require more rigid constructs (ORIF-plus, hindfoot fusion) and longer protection; non-operative management of unstable fractures is discouraged. Primary arthrodesis considered for severe destruction or failed fixation	Diabetic foot and ankle fractures show higher rates of infection, non-union and amputation than in non-diabetics (e.g. ≈2.8× infection, 6.5× non-union, 7.4× amputation in some series). Non-operative treatment of displaced ankle fractures in diabetics associated with ~ 21× higher complication risk than early ORIF. Calcaneal fractures: wound complications reported in 78% of DM patients vs. 22.7% in non-diabetics
Diabetes and healing outcomes in lower extremity fractures: a systematic review Gortler et al. (2018)	Systematic review	8 studies; 4671 pts (483 DM, 4188 non-DM). Fracture types: ankle (5 studies), tibia (1), pilon (1), hip (1).	Diabetes is a major risk factor for adverse healing: malunion, infection and re-operation. Insulin-dependence, younger diabetic age and poor compliance were associated with higher infection and complication rates in some ankle cohorts.	All fractures were surgically treated	Ankle fracture data contributed to overall increased risks in diabetic patients: Malunion OR 6.27 Infection OR 3.19 Deep infection OR 4.00 Re-operation OR 5.45 In below-knee subgroup (including ankle): Non-union OR 7.30 for diabetic In one ankle series, non-operative treatment led to more malunions, whereas operative fixation reduced deformity at the cost of more wound complications
The impact of diabetes on outcomes for tibiotalocalcaneal arthrodesis: a systematic review of available comparative studies Talaski et al. (2025)	Systematic review	4 studies; 162 patients total: 81 with diabetes, 81 without; mean age ~ 58 years; mean follow-up ~ 35 months. Diabetic patients commonly had peripheral neuropathy, PAD, prior ulcers and renal disease	Diabetic patients commonly had peripheral neuropathy, peripheral arterial disease, prior ulcers and renal disease, all linked to increased wound and limb-loss risk in these cohorts. Poor glycaemic control (elevated HbA1c) noted in related TTC series as a potential contributor to complications and fusion failure.	All studies evaluated tibiotalocalcaneal arthrodesis using retrograde intramedullary nails for hindfoot/ankle pathology. TTC fusion used as salvage or primary treatment in high-risk patients (e.g. Charcot, severe deformity, failed fixation), providing a rigid, load-sharing construct.	Fusion rates after TTC arthrodesis were high and generally similar in diabetics and non-diabetics Diabetic patients had 8x higher superficial wound infection and more wound complications overall. Deep infection and non-union rates were broadly comparable when robust TTC constructs were used. Limb-salvage rate 95%, 5% amputation rate
Bone quality and fracture healing in Type 1 and Type 2 diabetes Henderson et al. (2019)	Narrative review	Summarises data from multiple human observational cohorts, clinical series and trials comparing diabetic and non-diabetic patients across various fracture sites, including lower-extremity and ankle surgery	Diabetes is a major risk factor for impaired fracture-healing, with systemic complications such as neuropathy and vascular disease contributing to higher infection and fixation-related problems. Poor glycaemic control (high HbA1c or inadequate perioperative glucose control) is associated with increased surgical site infection and worse outcomes after orthopaedic procedures, including ankle fixation.	N/A	Diabetes associated with higher rates of delayed union, non-union, surgical site infection and fixation failure compared with non-diabetic patients. Each 1% increase in HbA1c increases the odds of surgical infection by ~ 1.6× in orthopaedic cohorts, including ankle surgery
Managing acute ankle and hindfoot fracture in diabetic patients Phyo et al. (2022)	Narrative review	Summarises multiple series of neuropathic and vasculopathic diabetics with ankle/hindfoot fractures; no pooled total. Includes cohorts with undisplaced fractures treated non-operatively and displaced/unstable fractures treated with ORIF or primary hindfoot fusion.	Peripheral neuropathy is the key risk factor, strongly linked to Charcot, deformity and amputation. PAD, poor glycaemic control, renal/cardiac disease and previous Charcot or ulceration associated with infection, wound problems, delayed/non-union and limb loss. Long-standing diabetes and poor compliance with non-weight-bearing increase failure of non-operative treatment.	Stable, undisplaced fractures: may be managed non-operatively in select diabetics with strict prolonged NWB casting and close radiographic monitoring Unstable/displaced fractures (especially neuropathic/PVD patients): require early operative fixation. Preferred constructs: rigid fixation (augmented ORIF/primary hindfoot fusion with IM nails/external frames), combined with optimisation of glycaemic and vascular status and prolonged protected weightbearing.	Diabetic ankle fractures have higher rates of wound breakdown, infection, non-union, Charcot and amputation compared to non-diabetics In neuropathic patients, undisplaced stable fractures can heal well non-operatively, but displaced fractures often progress to deformity, Charcot and need for fusion if not surgically stabilised. Lovy cohort: non-operative treatment of 20 displaced fractures in diabetics had 21× higher odds of complications than early ORIF in 8 patients, with 100% complications after failed casting vs. 12.5% after planned early fixation. Primary hindfoot/ankle arthrodesis series in complicated diabetics report limb-salvage rates ≈ 80–90%, with acceptable function despite frequent fusion non-union

### Risk of Bias

**Fig. 2:**
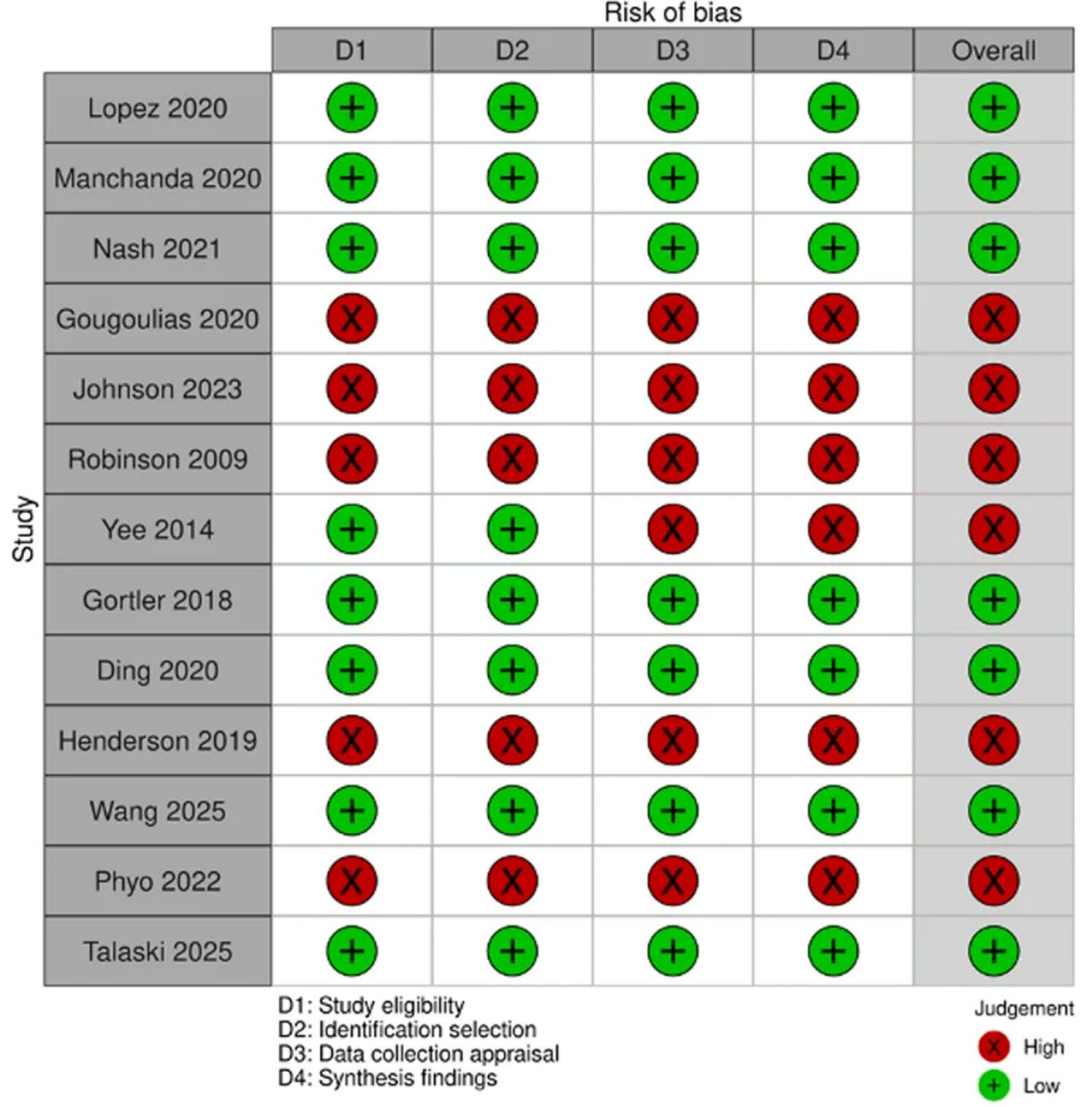
ROBIS traffic light plot showing risk of bias assessment for included reviews. Each row represents one of the 13 included reviews, with assessments across four domains: study eligibility criteria, identification and selection of studies, data collection and study appraisal, and synthesis and findings. Green indicates low concern; red indicates high concern

**Fig. 3:**
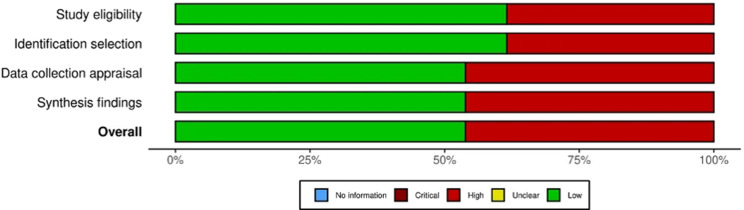
ROBIS summary bar chart showing overall risk of bias across domains. Stacked bar chart displaying the proportion of reviews rated at low risk (green) versus high risk (red) of bias for each ROBIS domain and overall assessment

Risk of bias for the 13 included reviews was assessed using the ROBIS tool (Figs. 2 and 3). Seven systematic reviews were judged at low concern across all four domains, reflecting clearly defined eligibility criteria, comprehensive multi-database searches, structured data extraction, formal quality appraisal and transparent synthesis methods [9, 16–21]. In contrast, six narrative or non-systematic reviews showed high concern in all domains because they lacked reproducible search strategies, explicit inclusion criteria, formal risk-of-bias assessment of primary studies, or systematic synthesis [13, 22–26]. Overall, approximately half of the secondary evidence base was rated at low risk of bias, with the remainder judged at high risk.

### Development of the MADRAS Scoring System

Based on the synthesised evidence from this review, we developed the Manchester Ankle Fracture Diabetic Risk Assessment Score (MADRAS), an evidence-weighted risk stratification tool for diabetic ankle fractures. Unlike the original AFDA, which assigned uniform weighting to risk factors based primarily on expert consensus, MADRAS assigns differential weighting based on the magnitude of effect observed across the included reviews. Points were assigned in proportion to observed effect sizes: risk factors with OR ≥ 10 received 3 points, those with OR 3–10 or complication rates exceeding 50% received 2 points, and those with OR < 3 received 1 point. Where no pooled OR was available, weighting was based on reported complication rates or expert clinical consensus, as detailed in Tables 2, 3 and 4. The MADRAS score functions as an evidence-informed, consensus-based clinical framework rather than a statistically derived predictive model. It was created through structured interpretation of effect sizes and risk estimates reported in the reviews included, combined with expert consensus from the author group, rather than through multivariable regression or patient-level data modelling [15].

The MADRAS framework comprises three domains (Tables 2, 3 and 4). Domain A addresses systemic host factors derived from the original AFDA. Peripheral neuropathy received the highest weighting (3 points) based on its identification as the single strongest predictor of complications, with ORs ranging from 12 to 15.49 across included reviews [18]. Peripheral arterial disease received 2 points based on complication rates exceeding 80% in patients with absent pedal pulses [18] and its role as a key component of complicated diabetes (overall OR 8.4) [16]. History of Charcot neuroarthropathy received 2 points, reflecting OR 5.1 for Charcot-related complications [16], which falls within the OR 3–10 threshold. Nephropathy, retinopathy, and diabetes duration greater than 10 years each received 1 point as markers of complicated diabetes status.

Domain B represents a novel addition addressing a key limitation identified in this review: the absence of local injury factors in the original AFDA. Fracture-dislocation or severe instability received 1 point. Open fractures or severe soft tissue compromise received 2 points, based on the 76.9% overall complication rate observed in affected cohorts [22]. Comminuted or pilon-variant patterns, or significant bone loss, received 2 points given the increased complexity requiring more than standard fixation.

Domain C addresses modifiable biological and patient factors. Poor glycaemic control (HbA1c > 8%) received 1 point, reflecting the evidence that each 1% HbA1c increase raises surgical site infection risk by 1.6 times [27]. Active smoking and poor compliance with non-weight-bearing protocols each received 1 point as modifiable factors associated with fixation failure.

The total MADRAS score (range 0–17) stratifies patients into four management categories (Table 5): low risk (0–2 points), where standard ORIF is appropriate with outcomes comparable to non-diabetics; moderate risk (3–5 points), where augmented fixation (‘ORIF Plus’) with supplemental stabilisation is recommended based on evidence showing reduced complication rates (5.7% vs. 20%) [18]; high risk (6 points), where primary arthrodesis or limb-salvage constructs such as TTC nailing or circular external fixation should be considered; and very high risk (≥ 7 points), where hindfoot nail fixation or circular external fixation is recommended, with reported limb-salvage rates of approximately 95% [21]. The management algorithm applies to fractures requiring operative intervention; non-operative management with strict non-weight-bearing and close radiographic monitoring remains appropriate only for stable, undisplaced fractures in low-risk (uncomplicated) diabetic patients, as detailed in the Discussion.

**Table 2:** Domain A: Systemic Host Factors. Risk factors derived from the original AFDA framework and validated by the current review, with evidence-based point allocations ranging from 1–3 points based on observed effect sizes

Risk Factor	Points	Rationale for Weighting	Supporting Evidence
Peripheral Neuropathy (Loss of protective sensation)	3	Identified as the “single strongest predictor” of complications; ORs ranging from 12 to 15.49 across reviews.	12/13 studies (López, Manchanda, Gougoulias, Grote, Yee, Robinson, Nash, Ding, Johnson, Phyo, Henderson, Talaski). Reported in 5/7 low bias and all 6 high-bias studies. OR range: 12–15.49 across reviews. OR ≈ 12 for surgical site infection; OR 15.49 for major complications (Nash et al.). Exceeds the OR ≥ 10 threshold, supporting 3 points.
Peripheral Arterial Disease (Vasculopathy/Absent pulses)	2	Complication rate of 83% with absent pedal pulses exceeds the 50% threshold for 2 points; critical ischaemia often precludes standard fixation.	10/13 studies (López, Gougoulias, Grote, Robinson, Nash, Ding, Johnson, Phyo, Henderson, Talaski). Reported in 4/7 low-bias and all 6 high-bias studies. Complications in approximately 83% of patients with absent pedal pulses (Robinson et al.), exceeding the 50% complication rate threshold for 2 points. OR 2.9 for amputation specifically (López et al.); PAD is also a key component of “complicated diabetes,” which carries an overall OR of 8.4 (López et al.).
History of Charcot Neuroarthropathy	2	Indicates severe susceptibility to deformity and bone failure.	7/13 studies (López, Gougoulias, Grote, Yee, Robinson, Johnson, Phyo). Reported in 1/7 low-bias and 5/6 high-bias studies. OR 5.1 for Charcot/malunion/non-union (López et al.); this was the only specific OR reported for this factor across the included reviews. Falls within the OR 3–10 threshold, supporting 2 points.
Nephropathy or Retinopathy	1	Markers of end-organ damage and “complicated” diabetes status.	10/13 studies (López, Manchanda, Gougoulias, Grote, Yee, Robinson, Ding, Johnson, Phyo, Talaski). Reported in 4/7 low-bias and all 6 high-bias studies. No standalone OR reported; consistently described as markers of end-organ damage and complicated diabetes status.
Duration of Diabetes > 10 years	1	Associated with accumulated microvascular damage.	3/13 studies (Yee, Robinson, Phyo). Reported in 0/7 low-bias and 3/6 high-bias studies. No specific OR reported; described as associated with accumulated microvascular damage and increased failure of non-operative treatment.

**Table 3:** Domain B: Local Injury Factors. New additions to the scoring system addressing fracture characteristics and soft tissue status, with point allocations of 1–2 points based on complication rates from the included reviews

Risk Factor	Points	Rationale for Weighting	Supporting Evidence
Fracture-Dislocation / Severe Instability	1	Displaced or unstable patterns mandate operative management. The OR of 21 cited in the supporting evidence relates to the comparison of non-operative versus operative treatment of displaced fractures, not to the independent effect of fracture severity on post-operative complications; 1 point is assigned to flag the injury pattern as a modifier requiring surgical attention.	4/13 studies (Grote, Nash, Johnson, Phyo). Reported in 1/7 low-bias and 3/6 high-bias studies. Non-operative management of displaced fractures associated with approximately 21× higher complication risk than early ORIF (Phyo et al., Johnson et al.).
Open Fracture or Severe Soft Tissue Compromise	2	Associated with very high infection and non-union rates (76.9% complications in affected cohorts, exceeding the 50% complication rate threshold for 2 points).	3/13 studies (Grote, Nash, Johnson). Reported in 1/7 low-bias and 2/6 high-bias studies. Overall complication rate of 76.9% reported in cohort with high proportion of open fractures and severe injury patterns (Grote et al.).
Comminuted / Pilon / Bone Loss	2	No specific OR reported in the included reviews. Assigned 2 points by expert consensus, reflecting comparable clinical severity to open fractures (which carry 76.9% complication rates) and the established requirement for fixation beyond standard ORIF.	Not directly reported as an isolated risk factor in the included studies. Inclusion based on expert consensus reflecting established clinical complexity requiring fixation beyond standard ORIF.

**Table 4:** Domain C: Biological and Patient Factors. Modifiable risk factors including glycaemic control, smoking status, and compliance, each allocated 1 point based on their association with fixation failure and complications

Risk Factor	Points	Rationale for Weighting	Supporting Evidence
Poor Glycaemic Control (HbA1c > 8%)	1	Review found each 1% HbA1c increase raises infection risk by 1.6×. >8% is a common threshold for “poor control”.	11/13 studies (López, Gougoulias, Grote, Yee, Robinson, Ding, Johnson, Phyo, Henderson, Talaski, Wang). Reported in 4/7 low-bias and all 6 high-bias studies. Each 1% increase in HbA1c raises surgical site infection odds by approximately 1.6× (Henderson et al.).
Active Smoking	1	Compounding vasoconstrictive risk factor.	Not directly reported as an isolated risk factor in the included studies. Inclusion based on expert consensus reflecting its established vasoconstrictive and wound-healing effects.
Poor Compliance / Inability to NWB	1	Non-compliance is a key driver of fixation failure in neuropathic patients.	3/13 studies (Nash, Gortler, Phyo). Reported in 2/7 low-bias and 1/6 high-bias studies. Poor compliance with non-weight-bearing consistently identified as a key driver of fixation failure and non-operative treatment failure.

**Table 5:** Updated Management Algorithm. Four-tier risk stratification based on total MADRAS score (0–17 points) with corresponding intervention strategies ranging from standard ORIF to hindfoot nail fixation or circular external fixation

Total Score	Risk Category	Suggested Intervention Strategy
0–2	Low Risk (Uncomplicated)	Standard ORIF. Treat similarly to non-diabetic patients + Close Monitoring.
3–5	Moderate Risk	Augmented Fixation (“ORIF Plus”). Rigid internal fixation with supplemental stabilisation (Fibula-Pro-Tibia).
6	High Risk (Complicated)	Hindfoot Nail Fixation / Primary Arthrodesis / Limb Salvage (TTC Nail / Circular External Fixation).
≥ 7	Very High Risk	Hindfoot Nail Fixation / Circular External Fixation.

It should be noted that MADRAS deliberately does not sub-classify certain variables, such as the severity of peripheral arterial disease, specific types of instability, or detailed pilon fracture configurations. The primary purpose of MADRAS is to stratify the diabetic comorbidity burden that modifies fracture outcomes, rather than to serve as a fracture classification system; established fracture classification systems already fulfil that role. Within this framework, Domain B differentiates between stable and unstable fracture patterns, and in patients with unstable fractures who also carry significant diabetic comorbidity, the current evidence supports augmented fixation or hindfoot nailing rather than standard constructs [18, 21]. It should also be acknowledged that non-diabetic risk factors, including age, sex, and body mass index, are relevant to surgical decision-making in ankle fractures; however, these factors are pertinent to all ankle fractures irrespective of diabetic status and are therefore not included as diabetes-specific variables within MADRAS. Clinicians should consider these alongside the MADRAS score when planning management. MADRAS serves as a prompt for clinicians to actively identify and consider diabetes-related risk factors during surgical planning, while recognising that patient-specific variations are likely, and that clinical judgment remains essential. Importantly, the management algorithm applies to fractures requiring operative intervention; MADRAS does not recommend non-operative treatment of unstable fractures in any risk category. Non-operative management with strict non-weight-bearing and close radiographic monitoring remains appropriate only for stable, undisplaced fractures in low-risk patients, consistent with the evidence summarised in this review. Although the proposed score requires prospective clinical validation, it is grounded in and supports the current evidence base. In addition, the proposed risk‑category cut‑offs and associated management strategies are intended to guide and support, not replace, individual clinician judgement and patient‑specific decision‑making.

## Discussion

Diabetic ankle fractures are among the most challenging injuries in foot and ankle care, with higher rates of complications and healthcare use compared to patients without diabetes [16–18]. Reviews show these injuries have greater risks of wound problems, deep infection, non-union, Charcot neuroarthropathy, and amputation. They are also often misdiagnosed or poorly managed, leading to longer hospital stays and more procedures [16–18, 23, 24]. There is still no widely accepted guideline, so treatment varies [17, 23]. The AFDA classification was proposed and developed to provide a structured tool based on clinical data and expert input [13]. However, it was created from a single centre’s experience and has not been widely validated. Since then, several systematic reviews, meta-analyses, and narrative reviews have been published [9, 16–18, 20, 21, 23, 24]. Advances in fixation strategies and understanding of disease pathology have occurred since. This review brings together that evidence to re-examine the AFDA framework and highlight areas for improvement.

A common finding in the reviews is the difference in outcomes between uncomplicated and complicated diabetes after ankle fractures. Patients with uncomplicated diabetes, meaning no neuropathy or major organ disease, tend to have complication rates similar to those without diabetes when given standard treatment and follow-up [17, 23, 24]. In contrast, complicated diabetes, which includes conditions like peripheral neuropathy, peripheral arterial disease (PAD), nephropathy, retinopathy, poor blood sugar control, or previous Charcot neuroarthropathy, leads to much worse outcomes [9, 17, 18, 23, 24]. López et al. found that advanced or complicated diabetes increases the risk of complications by about eight times compared to uncomplicated cases [16], and other reviews report similar risks when neuropathy or vascular disease is present [18, 23, 24]. This distinction aligns with the original AFDA idea that diabetes should be viewed as a range of risks, not just a single factor [13]. Our results support the AFDA view that low-risk, uncomplicated patients can often be treated like non-diabetic patients, while complicated cases need a more careful, intensive approach. This highlights the need for further refinement of any score for risk stratification.

All reviews highlight the same health conditions as major predictors of poor outcomes, matching the AFDA risk factors. Peripheral neuropathy is the strongest predictor, with PAD also critical—one study found complications in 10 of 12 patients with absent pedal pulses [18]. Other factors including nephropathy, retinopathy, long-term diabetes, and previous Charcot are consistently linked to complications [17, 18, 23, 24]. Together, these findings support the AFDA principle that cumulative risk, not diabetes alone, should guide treatment decisions.

Choosing between surgical and non-surgical management of diabetic ankle fractures is a key factor in patient outcomes. Most reviews agree that non-surgical treatment is not safe for most diabetic ankle fractures if the bone is out of place or unstable. In complicated diabetes, conservative treatment often leads to quick loss of alignment, deformity, and high rates of infection and amputation [18, 23–26]. Phyo and Wee, using data from Lovy et al., found a 21-fold increase in major complications when displaced fractures in diabetics were treated without surgery, and all patients had complications if casting failed [26]. Nash, Johnson, and Robinson also report very high rates of deformity, repeated ulcers, and limb loss with non-surgical care for unstable fractures [18, 23–25]. Gortler et al. found more malunions in diabetic ankles treated without surgery, and Gougoulias et al. advise against non-surgical care except for the most stable fractures [9, 23]. Only stable, undisplaced fractures in uncomplicated diabetes, with strict non-weight-bearing and close X-ray checks, seem suitable for conservative treatment [9, 17]. These results suggest that non-surgical care should be avoided in moderate- to high-risk diabetic ankles and only used for carefully chosen, stable injuries in low-risk patients.

Given these challenges, surgery is the main treatment, with fixation strength matched to risk level [9, 13, 16–18, 21, 23, 24]. For uncomplicated diabetics, standard ORIF achieves results comparable to non-diabetics; Manchanda et al. reported 14–15% infection, 4% non-union, and 98% limb-salvage rates [17]. For higher-risk patients, Nash and colleagues found fewer mechanical failures with augmented fixation [18]. For the highest-risk patients, primary arthrodesis is supported as a limb-salvage strategy [9, 16–18, 21], and circular external fixation (Ilizarov-type frames) offers an alternative when internal fixation is contraindicated or bone quality is severely compromised. These results support graduated treatment: standard ORIF for low risk, stronger fixation (ORIF Plus) for moderate risk, and early fusion, TTC super-constructs, or circular external fixation for the highest scores.

These points suggest that the current AFDA model may underestimate risk by not adequately weighting local injury characteristics, which could limit its usefulness. To address these limitations, we developed MADRAS, which includes fracture type and soft-tissue condition as separate, weighted factors within a new local Injury domain. MADRAS addresses AFDA’s limitations through three key advances: first, proportional weighting of risk factors based on observed effect sizes, ensuring the strongest predictors contribute most to the score; second, incorporation of local injury factors as a distinct domain (Domain B), recognising that fracture severity independently predicts complications; and third, a four-tier management algorithm that includes an intermediate ‘ORIF Plus’ category for moderate-risk patients, reflecting contemporary evidence for augmented fixation techniques.

Another important theme is patient behaviour and healing capacity, not formally included in the original AFDA [13]. Non-compliance with non-weight-bearing, smoking, and social factors are linked to poor outcomes [9, 18]. Diabetes roughly doubles healing time and non-union risk [11], though Wang et al. found early protected weight-bearing may be safe in selected low-risk patients [19]. Bringing these findings together, MADRAS incorporates these factors through Domain C (Biological and Patient Factors), which includes glycaemic control, smoking status, and compliance as weighted criteria alongside the systemic and injury-related domains.

This study has several strengths. It is the first systematic review of reviews focused on diabetic ankle fractures and the AFDA framework—the only currently available framework, bringing together ten years of secondary evidence [9, 13, 16–21]. By combining systematic, meta-analytic, and narrative reviews, it offers a broader view than any single primary study [9, 17–25]. However, important limitations remain. First, the synthesis of the evidence is limited by the differences in how key outcomes such as infection, union, and amputation are defined across studies. These differences not only limit direct comparisons but also make formal meta-analysis difficult [9, 13, 17–21]. Also, because this is a review of secondary literature, it was not possible to combine primary patient data. Some reviews included the same primary studies, which complicates the interpretation of key outcomes. Including both systematic and narrative reviews, which may overlap in primary studies, introduces heterogeneity and the risk of double-counting that could influence the interpretation of the aggregated data. With a relatively small number of included reviews (*n* = 13), this overlap may lead to overrepresentation of certain primary datasets and could confound the conclusions drawn. To mitigate this, MADRAS weightings were based on the consistency of risk factor identification across independent reviews rather than on aggregation of individual study statistics, and the supporting evidence columns in Tables 2, 3 and 4 transparently report how many and which reviews informed each factor. These limitations highlight the need for future research that directly links patient-level data across studies and uses standardised definitions for outcomes and risk factors. Such analyses would enable more accurate comparisons, better risk stratification, and the development of robust, evidence-based clinical guidelines for diabetic ankle fracture management. The evidence here, like that behind the original AFDA, is mostly observational and does not allow for setting precise score thresholds or ideal weighting [13, 16–18, 23, 24].

A further limitation is the risk of bias among the included studies. As reported in the results, six of the 13 included reviews were rated at high risk of bias across all ROBIS domains, meaning approximately half of the evidence base underpinning MADRAS comes from narrative reviews lacking reproducible search strategies, formal quality appraisal, and systematic synthesis. However, the risk factors carrying the highest weight for MADRAS—peripheral neuropathy and peripheral arterial disease—were consistently reported across both high and low bias studies, supporting their inclusion. Where risk factors were described primarily in high-bias reviews, their weightings should be interpreted with greater caution. This reinforces the view that MADRAS is an evidence-informed clinical framework and hence prospective validation is essential before widespread clinical adoption.

The proposed MADRAS scoring system, while derived from synthesised evidence across multiple reviews, has not yet been prospectively validated. The assigned weightings are based on relative effect sizes from heterogeneous studies with varying outcome definitions, and the optimal score thresholds for management stratification require external validation in prospective multicentre cohorts before widespread clinical adoption can be recommended.

## Conclusion

Despite these limitations, the findings of this review provide an updated, higher-level evidence base for a risk-stratified approach to diabetic ankle fracture management and reinforce AFDA’s view that outcomes are shaped by both systemic and local risk factors, not just diabetes alone [9, 13, 16–18, 20, 23, 24, 27]. The consistent evidence that these factors predict wound breakdown, fixation failure, deformity, and amputation support continued use of a cumulative risk score to guide surgical decisions [9, 17, 18, 20, 23, 24, 27]. At the same time, this review identifies new areas, injury severity, patient compliance, and healing capacity, which we have incorporated into the proposed MADRAS scoring system. Future research should prospectively validate MADRAS across multiple centres to determine whether the evidence-based weightings translate into improved clinical decision-making and patient outcomes. Until such prospective external validation is available, MADRAS should be regarded as a decision-support tool rather than a definitive, outcome-predictive score. It is hoped that this paper will focus clinicians’ attention on the importance of systematic risk stratification for patients with ankle fractures and diabetes.

## Data Availability

All data generated or analysed during this study are included in this published article.

## Abbreviations

AFDA: Adelaide Fracture in the Diabetic Ankle
AGEs: Advanced Glycation End Products
BMI: Body Mass Index
CI: Confidence Interval
DM: Diabetes Mellitus
HbA1c: Glycated Haemoglobin
IDDM: Insulin-Dependent Diabetes Mellitus
MADRAS: Manchester Ankle Fracture Diabetic Risk Assessment Score
NIDDM: Non-Insulin-Dependent Diabetes Mellitus
NWB: Non-Weight-Bearing
OMAS: Olerud-Molander Ankle Score
OR: Odds Ratio
ORIF: Open Reduction Internal Fixation
PAD: Peripheral Arterial Disease
PVD: Peripheral Vascular Disease
ROBIS: Risk of Bias in Systematic Reviews
ROBVIS: Risk of Bias Visualisation
ROM: Range of Motion
SMD: Standardised Mean Difference
SSI: Surgical Site Infection
TTC: Tibiotalocalcaneal
